# A multicohort proteomics-based machine learning score (MASHPredict) for noninvasive diagnosis and long-term prognostication of metabolic dysfunction-associated steatohepatitis

**DOI:** 10.3389/fimmu.2026.1849699

**Published:** 2026-10-15

**Authors:** Xu Hu, Fanlai Meng, Wei Zhou, Shun Li, Yujie Gao, Yali Zhang, Jie Zhang, Lianhua Kong, Guokun Liu, Xiaohong Shi, Jianbo Xu, Qing Zhang, Wen Hu

**Affiliations:** 1 Department of Endocrinology and Metabolism/Clinical Nutrition, the Affiliated Huai’an Hospital of Xuzhou Medical University, Huai’an, China; 2 Department of Pathology, Nanjing Drum Tower Hospital Group Suqian Hospital, Suqian, China; 3 Department of Endocrinology and Metabolism, Huai’an 82 Hospital, Huai’an, China; 4 Department of Neurology, Nanjing Drum Tower Hospital, the Affiliated Hospital of Medical School, Nanjing University, Nanjing, China; 5 Department of Medical Insurance, the First Affiliated Hospital of Nanjing Medical University, Nanjing, China; 6 Department of Nursing, the Affiliated Huai’an Hospital of Xuzhou Medical University, Huai’an, China; 7 Department of Hepatobiliary Surgery, the Affiliated Huai’an NO.1 People’s Hospital of Nanjing Medical University, Huai’an, China; 8 Department of Hepatology, Huai’an No. 4 People’s Hospital, Huai’an, China

**Keywords:** diagnosis, machine learning, metabolic dysfunction-associated steatohepatitis, plasma proteomics, prognosis

## Abstract

**Background:**

Metabolic dysfunction-associated steatohepatitis (MASH) can progress to cirrhosis and hepatocellular carcinoma, yet the invasiveness of liver biopsy limits its clinical utility. Accurate noninvasive diagnostic tools are essential for improved patient risk stratification.

**Methods:**

A machine learning study was conducted using plasma proteomics from 142 participants (83 MASH cases, 59 controls) recruited from a Chinese hospital, randomly divided into training and validation cohorts (7:3 ratio). Key proteins were identified via feature selection using Least Absolute Shrinkage and Selection Operator regression and the Boruta algorithm. Ten machine learning models were developed to create the MASHPredict score, with external validation in two test cohorts. Prognostic evaluation focused on liver-related events, atherosclerotic cardiovascular disease (ASCVD)-related mortality, and all-cause mortality.

**Results:**

MASHPredict demonstrated robust diagnostic performance, with Area Under the Curve (AUC) values of 0.83 (95% CI: 0.74–0.92) in the training cohort, 0.80 (0.66–0.94) in the validation cohort, 0.79 (0.67–0.91) in test cohort 1, and 0.79 (0.76–0.83) in test cohort 2. It outperformed established noninvasive tests, including the Fibrosis-4 Index, AST to Platelet Ratio Index, AST/ALT Ratio, and NAFLD Fibrosis Score. High-risk individuals exhibited significantly increased hazards for liver-related mortality (HR = 8.18), MASH (HR = 12.07), cirrhosis (HR = 3.37), liver cancer (HR = 3.11), ASCVD-related mortality (HR = 1.64), and all-cause mortality (HR = 1.42) (all p < 0.001).

**Conclusion:**

The MASHPredict score is a validated, noninvasive diagnostic tool with broad applicability. Its superior performance in diagnosing MASH and prognosticating liver-related outcomes supports its potential role in early detection, risk stratification, and clinical decision-making.

## Introduction

Metabolic dysfunction associated steatotic liver disease (MASLD) has risen progressively over recent decades. Currently, this condition affects approximately 38% of individuals worldwide (1, 2). Metabolic dysfunction-associated steatohepatitis (MASH) represents a progressive form of MASLD, posing significant clinical and economic challenges worldwide (3, 4). The disease burden arises from the potential progression to advanced fibrosis, cirrhosis, and hepatocellular carcinoma (5, 6). Patients with MASLD are at heightened risk for developing type 2 diabetes, atherosclerotic cardiovascular disease (ASCVD), and chronic kidney disease (7–9). Those with MASH and hepatic fibrosis are particularly vulnerable to these comorbidities, as well as certain extrahepatic malignancies (7–9). Given the severe clinical consequences, early detection and diagnosis of MASH are critical.

Liver biopsy remains the gold standard for diagnosing MASH (10). However, its clinical applicability is limited by several factors, including invasiveness, low patient acceptability, high costs, and the potential for interobserver variability. The procedure also carries inherent risks, with approximately 50% of patients experiencing postprocedural pain and life-threatening hemorrhage occurring in 0.6% to 1% of cases (11).

The development of accurate noninvasive tests (NITs) with strong diagnostic performance is essential for improving MASH detection across diverse populations. Such tests could reduce dependence on liver biopsy while enabling effective patient stratification for therapeutic interventions. Recent advancements in technology have highlighted proteomics—the comprehensive profiling of protein expression—as a promising strategy for identifying circulating biomarkers. These blood-based markers may offer insights into disease mechanisms and aid in early MASH detection (12).

In this study, machine learning techniques were employed to develop and validate a proteomics-based prediction score, referred to as the MASHPredict score, for noninvasive MASH assessment across varied ethnic populations. The study also evaluated the score’s ability to predict liver-related outcomes, ASCVD-related mortality, and all-cause mortality.

## Methods

### Study design

This study was conducted in two phases. The primary objective of phase one was to develop and validate the MASHPredict score for MASH diagnosis using plasma proteomics. Phase two aimed to evaluate the score’s ability to predict liver-related outcomes, ASCVD-related mortality, and all-cause mortality.

### Participants

Participants were recruited from the First Affiliated Hospital of Nanjing Medical University between August 2023 and July 2024 and were randomly assigned to training and validation groups with a 7:3 split. Patients with MASLD attended the Infectious Disease, General Surgery, or Bariatric and Metabolic Surgery departments. Eligibility required histologically confirmed liver biopsy, while exclusion criteria included viral hepatitis, established cirrhosis, and prior hepatic or other malignancies. Written informed consent was obtained from all eligible participants. The study protocol was approved by the Institutional Ethics Committee and conducted in accordance with the principles of the Declaration of Helsinki. All plasma samples were stored at −80 °C, with ≤2 freeze–thaw cycles before proteomic analysis. Samples with obvious hemolysis or lipemia were excluded to avoid interference with protein quantification. All specimens were processed in a single batch for proteomics to minimize technical variation and ensure reproducibility. Test cohort 1 was sourced from a previous Israel-based study with predefined eligibility criteria (13). Test cohort 2 consisted of UK Biobank participants who had both magnetic resonance imaging-proton density fat fraction (MRI-PDFF) measurements and proteomic profiling. These two cohorts were served as population-based external validation. The prognostic cohort, also from the UK Biobank, included individuals with comprehensive follow-up data. Both test cohort 2 and the prognostic cohort excluded participants with viral hepatitis, pre-existing cirrhosis, or prior hepatic or extrahepatic malignancies.

### Liver histology

Paraffin-embedded liver sections underwent standard hematoxylin and eosin (H&E) staining and Masson trichrome staining. Two experienced pathologists, blinded to group allocation, independently assessed histopathological features using the NASH Clinical Research Network scoring system (14).

### Outcome definitions and other definitions

In the training, validation, and test cohort 1, histological evidence of MASH was confirmed when the non-alcoholic fatty liver disease activity score (NAS) reached 4 or higher, with subscores of at least 1 for all three components: steatosis, hepatocyte ballooning, and lobular inflammation (15). For test cohort 2, diagnostic criteria included MRI-PDFF > 5.5% combined with iron-corrected T1 mapping (cT1) values > 800 ms (16). Prognostic assessment in the follow-up cohort focused on primary endpoints, including liver-related outcomes, ASCVD-related mortality, and overall mortality. Detailed definitions of events are provided in **Supplementary Table 1**.

### Modeling strategy

In the training cohort, Least Absolute Shrinkage and Selection Operator (LASSO) regression and the Boruta algorithm (17) were first applied to identify key variables associated with MASH. Ten predictive models were then constructed using the caret R package (18), which included K-Nearest Neighbors (KNN), Gradient Boosting Machine (GBM), Recursive Partitioning and Regression Trees (RPART), Neural Network (NNET), Support Vector Machine (SVM), AdaBoost, Random Forest (RF), Extreme Gradient Boosting (XGBoost), Generalized Linear Model with Elastic Net Regularization (GLMNET), and Partial Least Squares (PLS). Hyperparameter optimization was performed using 10-fold cross-validation combined with grid search. The PLS algorithm exhibited the best predictive performance in the validation cohort and was selected to construct the MASHPredict score, with grid search identifying optimal performance at ncomp = 1. SHapley Additive exPlanations (SHAP) values, visualized through the shapviz R package (19), were used to interpret the decision-making process of the optimal algorithm.

### Plasma proteomics

For each sample, 100 μL of plasma was enriched for low-abundance proteins using commercial human plasma high-abundance depletion magnetic beads (4 μL, pre-washed twice with washing buffer) with a 2-hour incubation at room temperature, followed by five 5-minute washing cycles to remove abundant plasma proteins. After magnetic separation, the captured low-abundance proteins were collected, and standard reduction and alkylation procedures were strictly implemented prior to digestion: dithiothreitol (DTT) was added for disulfide bond reduction, followed by iodoacetamide incubation to block free sulfhydryl groups and avoid disulfide reformation. Enriched proteins were digested with trypsin according to an in-solution protocol. C18 cartridges were used to desalt the resulting peptides. Following lyophilization, we reconstituted them in 20 μL of 0.1% formic acid. Peptide concentrations were measured through absorbance at 280 nm. Subsequently, iRT standard peptides were added to each sample before analysis.

Data independent acquisition (DIA) was conducted using a Vanquish Neo system (Thermo Fisher Scientific) connected to an Astral mass spectrometer. Peptide separation was achieved on a C18 column (1.9 μm, 150 μm \* 15 cm) through a linear gradient system. Mobile phase A contained 0.1% formic acid in water, whereas mobile phase B consisted of 0.1% formic acid in 80% acetonitrile. MS1 parameters comprised a scan range from 380 to 980 m/z, with 240, 000 resolution at 200 m/z. The normalized AGC target was set at 500%, and the maximum ion trap (IT) was 5 ms. For MS2 analysis, we employed 299 DIA windows with 2 m/z isolation. The HCD collision energy was 25 eV, normalized AGC target was 500%, and maximum IT was 3 ms.

We processed DIA data using DIA-NN software with trypsin digestion, allowing a maximum of one missed cleavage. Carbamidomethylation served as the fixed modification, whereas oxidation (M) and acetylation (protein N terminus) were set as variable modifications. A 1% false discovery rate (FDR) threshold was applied at the protein level. Differential analysis of plasma proteomics data was performed using the limma R package (20). QC samples were prepared by pooling equal volumes of all study plasma samples. All proteomic data were acquired in two analytical batches, and batch effects were corrected during data normalization. A coefficient of variation (CV) threshold of <20% for QC proteins was applied to assess quantitative reproducibility, ensuring robust and consistent proteomic results.

### Enrichment analysis

Pathway enrichment was performed using two methods: Over-Representation Analysis (ORA) *via* Metascape (21) and Gene Set Enrichment Analysis (GSEA) with the clusterProfiler R package (22).

### Single-nuclei RNA sequencing data analysis

snRNA-seq was performed on liver biopsies from patients diagnosed with MASLD (3 samples), MASH (5 samples), MASH with cirrhosis (4 samples), and end-stage disease due to MASLD progression requiring transplantation (6 samples). Healthy liver samples from donors (4 samples) served as controls and were processed following stringent quality control protocols (23). These data were derived from a previously published study and were processed in accordance with the quality control criteria established in that study. After quality control, 80, 698 cells were selected for downstream analysis using the Seurat R package (24). Gene expression levels were normalized using the LogNormalize method with a scale factor of 10, 000. The top 2, 000 highly variable genes were identified, scaled, and subjected to principal component analysis (PCA). Batch effects were corrected using the Harmony R package (25). All subsequent analyses, including NormalizeData, FindVariableFeatures, ScaleData, RunPCA, FindNeighbors, FindClusters, and RunUMAP, were conducted using the Harmony and Seurat packages (24, 25).

### Statistical analysis

All proteomic data underwent log2-transformation and Z-score normalization before analysis. Continuous variables were expressed as mean and standard deviation, whereas categorical variables were reported as frequency and percentage. We used Pearson or Spearman correlation to examine the relationships among variables. Receiver Operating Characteristic (ROC) curve analysis was performed to determine diagnostic performance of the MASHPredict score and comparator indices. Optimal thresholds derived from ROC analysis were applied to classify participants into high-risk and low-risk groups across cohorts. We performed Decision Curve Analysis (DCA) to evaluate net clinical benefit at various threshold probabilities. Kaplan-Meier methods were employed to assess survival outcomes. Cox proportional hazards regression models were adjusted for age, sex, BMI, diabetes status, and established noninvasive fibrosis markers including FIB-4, APRI, and NFS. A p value less than 0.05 was considered statistically significant. R software version 4.4.2 was used for all statistical analyses.

## Results

### Plasma protein landscape in MASH

The study workflow is illustrated in **Figure 1**. Proteomic profiling was performed on 142 samples, consisting of 83 MASH cases (58%) and 59 non-MASH controls (42%) (**Figure 2A**). Differential analysis identified 413 upregulated proteins (log fold change [logFC] > 0, p < 0.05) and 60 downregulated proteins (logFC < 0, p < 0.05) in MASH samples (**Figure 2B**; **Supplementary Table 2**). The top 20 proteins ranked by absolute logFC and the top 20 proteins with the lowest p-values are presented in **Figures 2B, C**, respectively. Two enrichment strategies were applied. ORA of the 413 upregulated proteins revealed enrichment in immune and metabolic pathways, such as neutrophil degranulation and carboxylic acid metabolic processes (**Figure 2D**). GSEA further confirmed activation of immune and metabolic pathways in MASH samples (**Figure 2E**). These findings align with the established pathophysiology of MASH, where metabolic dysregulation and chronic inflammation are key drivers of disease progression (26, 27).

**Figure 1:**
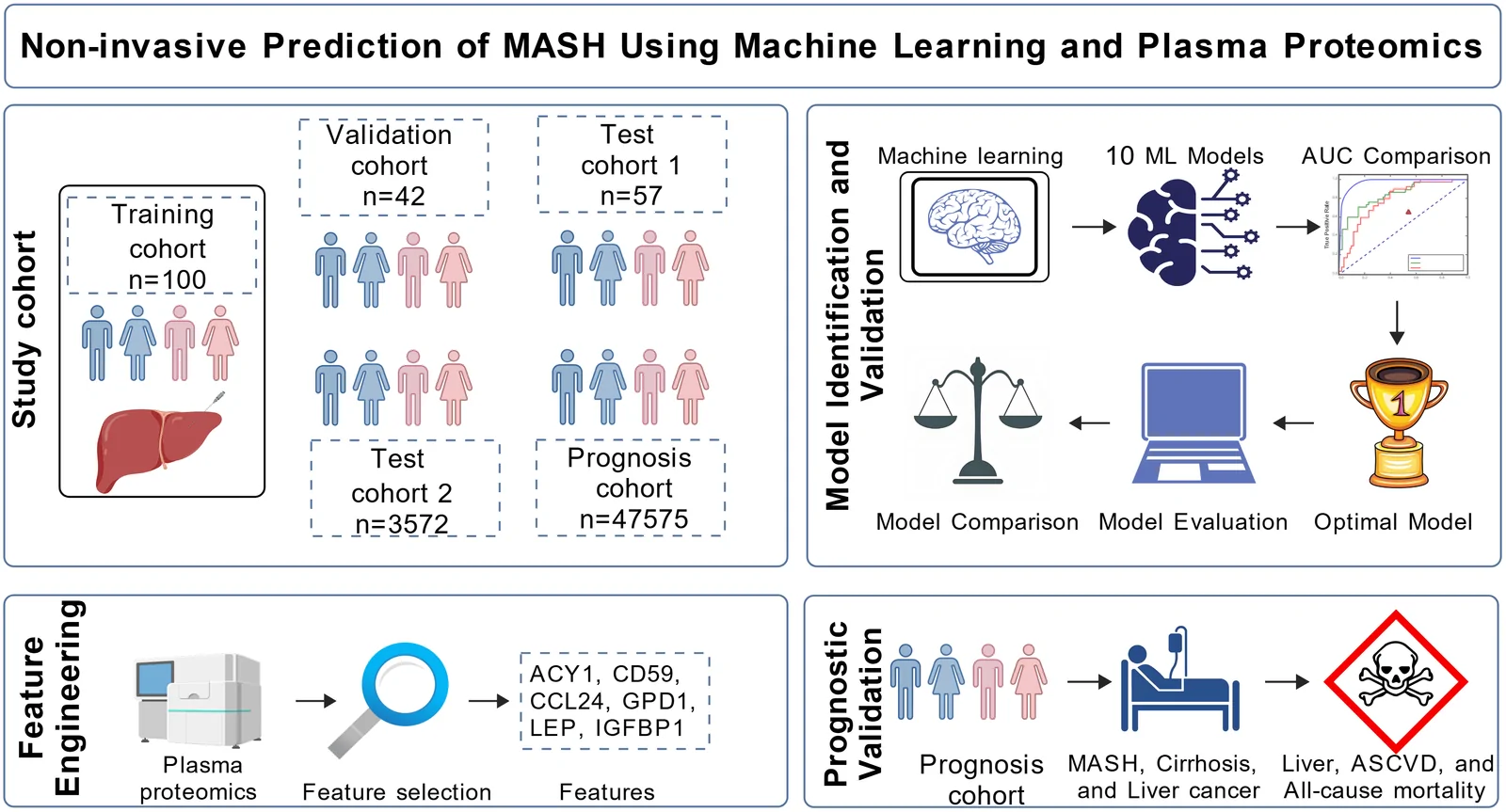
Schematic representation of the overall workflow employed in the present study. The workflow included (1) multi-cohort design with training (n=100), validation (n=42), test (n=57 and n=3572), and prognostic (n=47, 575) cohorts; (2) plasma proteomics profiling and feature selection of six core proteins (ACY1, CD59, CCL24, GPD1, LEP, IGFBP1); (3) machine learning model comparison and selection of the optimal PLS model based on validation AUC; and (4) diagnostic and prognostic validation of the MASHPredict score for MASH diagnosis and long-term mortality risk stratification.

**Figure 2:**
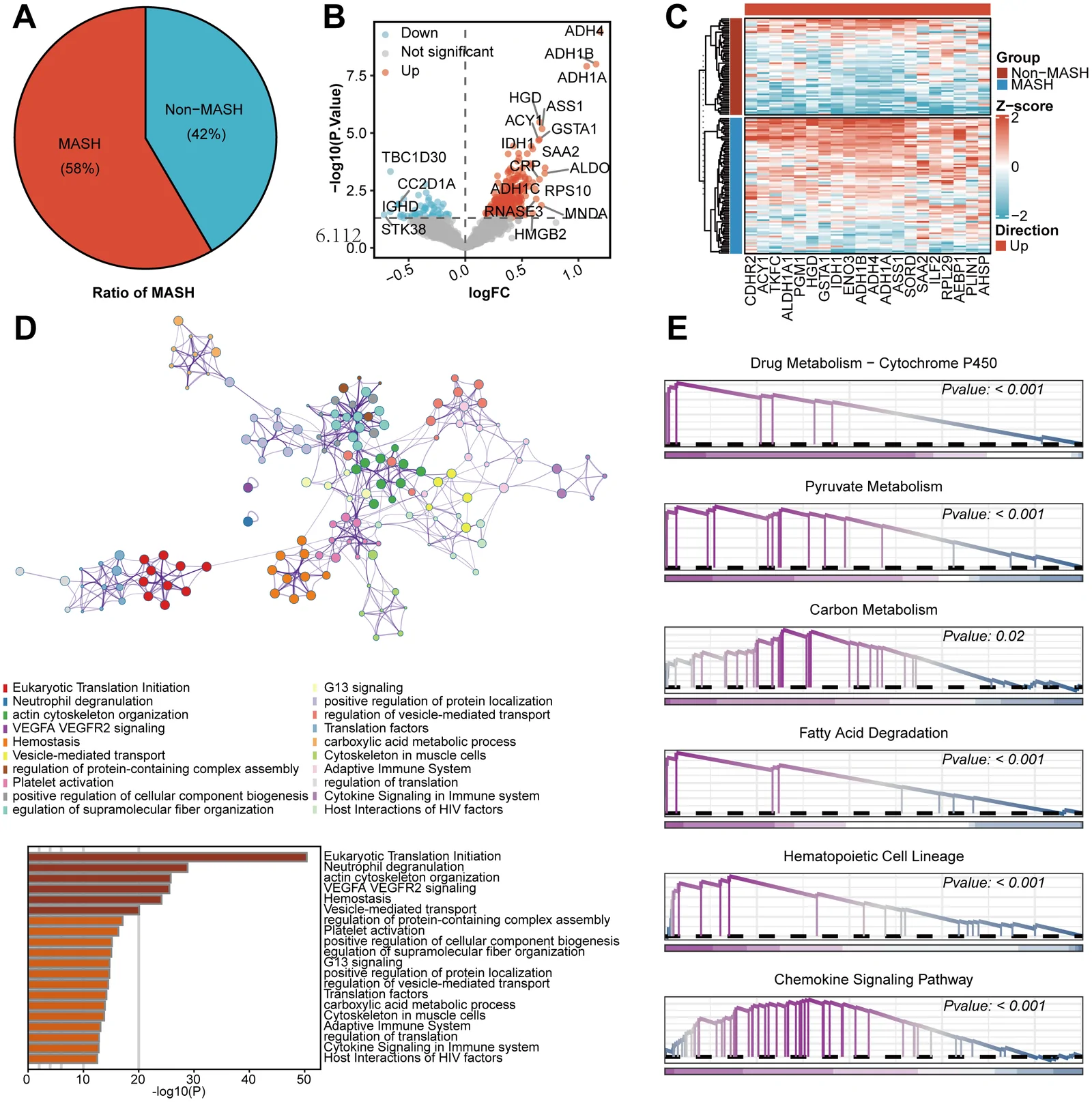
Plasma protein landscape in metabolic dysfunction-associated steatohepatitis (MASH). **(A)** Pie chart illustrating the distribution of 142 plasma samples, comprising 83 MASH cases (58%, red) and 59 non-MASH controls (42%, blue). **(B)** Differential protein expression between MASH and non-MASH samples. Volcano plot showing the relationship between fold change (log Fold Change, x-axis) and statistical significance (-log10 P value, y-axis) for all detected proteins. The top 20 proteins ranked by absolute logFC are labeled. **(C)** Heatmap visualization of top differentially expressed proteins. Z-score normalized expression levels of the top 20 proteins with the lowest p-values across all samples. **(D)** Protein-protein interaction network and pathway enrichment analysis. Upper panel: Network visualization of upregulated proteins showing functional clustering, with nodes colored by biological process categories as indicated in the legend. Lower panel: Horizontal bar chart displaying over-representation analysis (ORA) results for the 413 upregulated proteins. **(E)** Gene Set Enrichment Analysis (GSEA) of metabolic and signaling pathways. Bar charts showing enrichment profiles for six key pathways: Drug Metabolism-Cytochrome P450, Pyruvate Metabolism, Carbon Metabolism, Fatty Acid Degradation, Hematopoietic Cell Lineage, and Chemokine Signaling Pathway.

### Development of MASHPredict score

The 142 samples were randomly split into training and validation sets in a 7:3 ratio. Baseline characteristics of each cohort are shown in **Table 1**. In the training set, differential expression analysis was first performed to select proteins with p < 0.05, followed by LASSO and Boruta algorithm screening (**Figures 3A, B**), which identified six key proteins: ACY1, CD59, CCL24, GPD1, LEP, and IGFBP1. Correlation analysis confirmed that these proteins were not multicollinear (all correlations < 0.7) (**Supplementary Figure 1A**). Subsequently, ten distinct machine learning algorithms were trained on the training set and their performance evaluated on the validation set. The PLS algorithm demonstrated the highest AUC for MASH prediction in the validation set (**Supplementary Figure 1B**; **Supplementary Table 3**). Furthermore, PLS demonstrated the best overall performance in our comparative evaluation (**Supplementary Table 3**), achieving the highest F1-score (0.72), and the lowest Brier score (0.217), as well as a well-balanced specificity (0.73) and sensitivity (0.75), indicating superior discrimination and calibration. Given these combined advantages, PLS was selected as the final algorithm for constructing the MASHPredict score. The MASHPredict score exhibited robust predictive performance across all cohorts, with AUC values of 0.83 (95% CI: 0.74-0.92) in the training set, 0.80 (0.66-0.94) in the validation set, 0.79 (0.67-0.91) in test cohort 1, and 0.79 (0.76-0.83) in test cohort 2 (**Figures 3C–F**). Notably, MASHPredict scores were significantly higher in MASH participants compared to non-MASH controls across all cohorts (all p < 0.01) (**Figures 3C–F**).

**Table 1:** Baseline characteristics.

Characteristics	Training cohort (n = 100)	Validation cohort (n = 42)	Test cohort 1 (n = 57)	Test cohort 2 (n = 3, 572)	Prognosis cohort (n = 47, 575)
Male (%)	38 (38%)	13 (31%)	33 (58%)	1, 681 (47%)	21, 843 (46%)
Age (years)	32.71 (10.83)	30.21 (10.96)	NA	54.52 (7.73)	56.82 (8.21)
BMI (kg/m²)	38.85 (6.87)	38.29 (6.12)	NA	26.39 (4.07)	27.46 (4.79)
AST (U/L)	31.64 (11.74)	34.72 (13.79)	NA	22.75 (13.85)	23.23 (13.70)
ALT (U/L)	47.95 (35.79)	40.91 (36.06)	NA	25.67 (10.47)	26.22 (10.64)
HbA1c (%)	5.93 (2.05)	5.58 (1.32)	NA	5.35 (0.45)	5.47 (0.62)
PLT (10^9^/L)	111.78 (25.87)	110.64 (22.26)	NA	250.03 (56.21)	250.98 (61.98)
MASH (%)	63 (63%)	20 (48%)	37 (65%)	164 (4%)	NA
Type 2 diabetes (%)	31(31%)	7(17%)	NA	NA	NA
Cardiovascular diseases (%)	0 (0%)	0 (0%)	NA	NA	NA
Chronic kidney disease (%)	0 (0%)	0 (0%)	NA	NA	NA

Data are presented as mean (SD) or n (%). BMI, body mass index; ALT, alanine aminotransferase; HbA1c, glycated hemoglobin; AST, aspartate aminotransferase; MASH, metabolic dysfunction–associated steatohepatitis.

**Figure 3:**
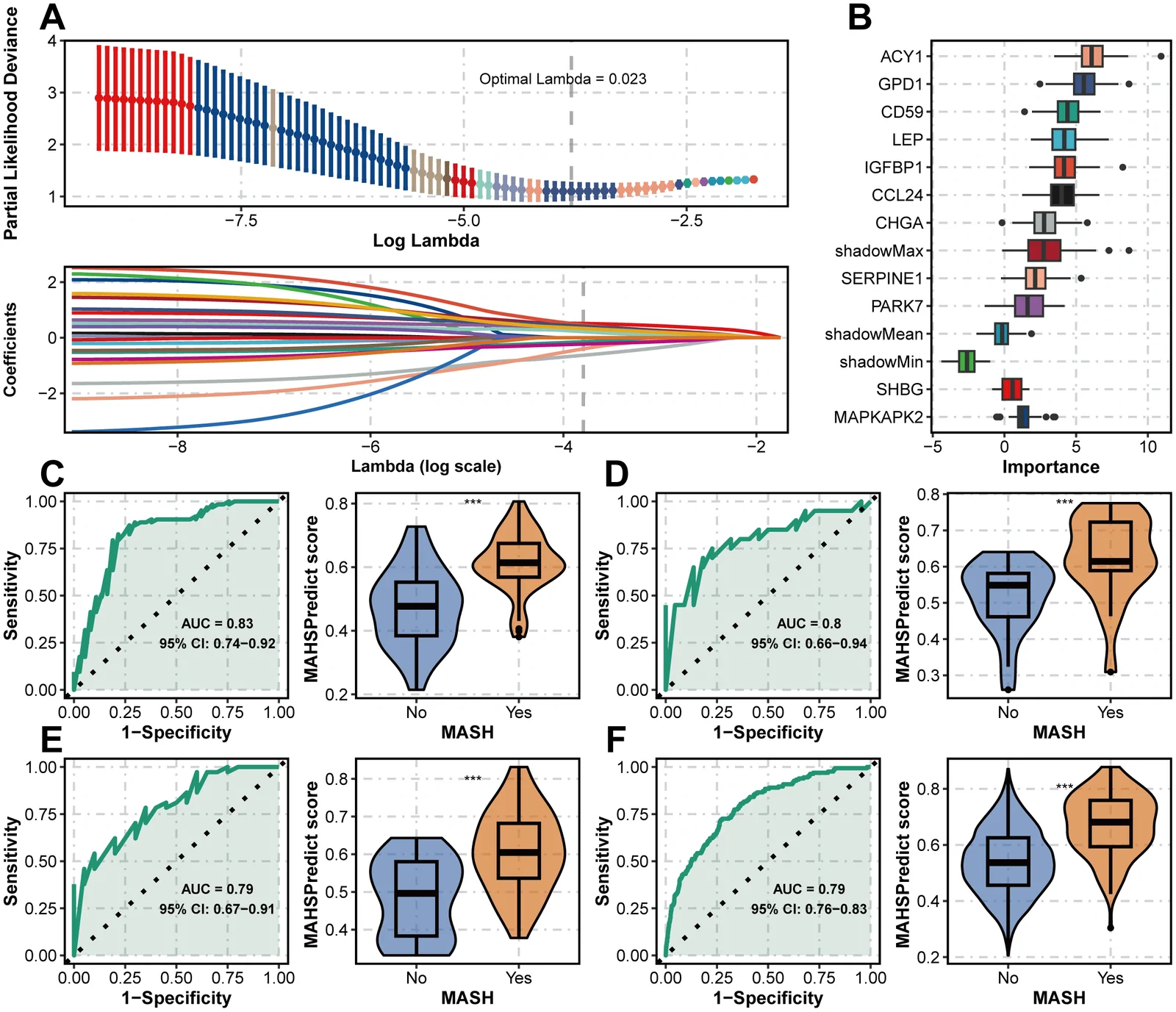
Development and validation of the MASHPredict score. **(A)** Least Absolute Shrinkage and Selection Operator regression analysis for feature selection. **(B)** Variable importance ranking from combined feature selection. **(C–F)**. ROC curve of MASHPredict score in predicting MASH across training cohort [**(C)**, left], validation cohort **(D)**, left], test cohort 1 [**(E)**, left], and test cohort 2 [**(F)**, left]. Comparative distribution of MASHPredict scores demonstrating clear differentiation between MASH and non-MASH across training cohort [**(C)**, right], validation cohort [**(D)**, right], test cohort 1 [**(E)**, right], and test cohort 2 [**(F)**, right]. *** denotes p < 0.001.

### Assessment of MASHPredict score

ROC curve analysis of the validation cohort established an optimal cutoff value of 0.58 for the MASH Predict score. This threshold was consistently applied across all subsequent cohorts for confusion matrix construction. The diagnostic performance of the MASH Predict score was systematically assessed through multiple validation phases. In the training cohort, the model achieved a sensitivity of 0.73 and specificity of 0.81 (**Figure 4A**; **Supplementary Table 4**). Validation in the validation cohort demonstrated a sensitivity of 0.75 and specificity of 0.73 (**Figure 4B**; **Supplementary Table 4**). In test cohort 1, the model attained a sensitivity of 0.62 and specificity of 0.75 (**Figure 4C**; **Supplementary Table 4**). Test cohort 2 exhibited a sensitivity of 0.80 and specificity of 0.63 (**Figure 4D**; **Supplementary Table 4**). Test cohort 2, which represented a general population sample with a MASH prevalence of only 5%, was particularly clinically relevant. In this cohort, the model achieved a negative predictive value (NPV) of 0.99, highlighting its exceptional ability to exclude MASH in populations with low disease prevalence. This remarkable NPV highlights the model’s potential as a noninvasive screening tool for clinical use.

**Figure 4:**
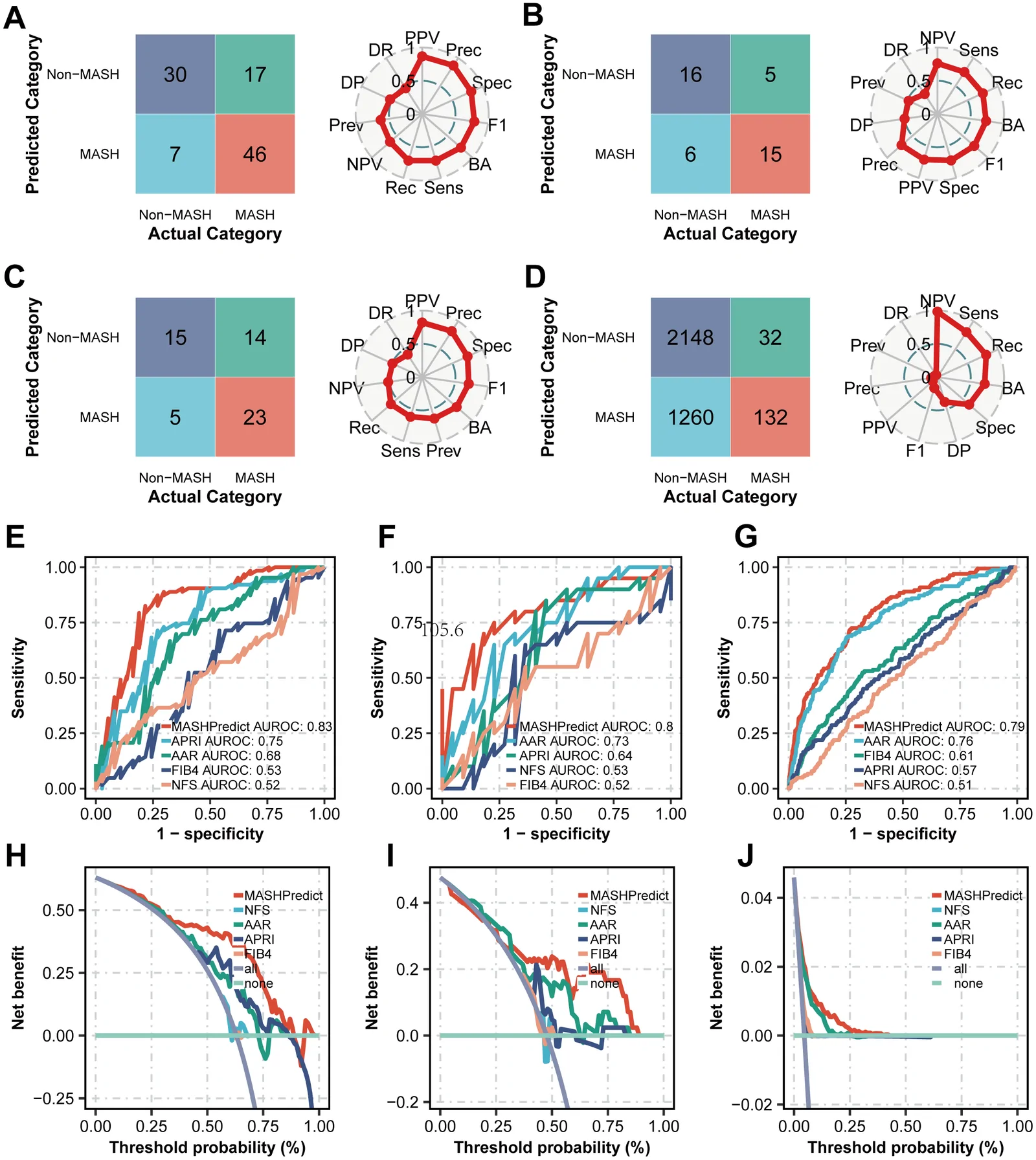
Assessment of the MASHPredict score. **(A–D)** Confusion matrices in the training cohort [**(A)**, left], validation cohort [**(B)**, left], test cohort 1 [**(C)**, left], and test cohort 2 [**(D)**, left]. Radar charts of classification metrics in the training cohort [**(A)**, right], validation cohort [**(B)**, right], test cohort 1 [**(C)**, right], and test cohort 2 [**(D)**, right]. **(E–G)** The receiver operating characteristic (ROC) curves of different non-invasive tests (NITs) in the training cohort **(E)**, validation cohort **(F)**, and test cohort 2 **(G)**. **(H–J)** The decision curve analysis (DCA) curves of different NITs in the training cohort **(H)**, validation cohort **(I)**, and test cohort 2 **(J)**. Spec, Specificity; Sens, Sensitivity; PPV, Positive Predictive Value; NPV, Negative Predictive Value; F1, F1 Score; Prec, Precision; Rec, Recall; Prev, Prevalence; DP, Diagnostic Probability; DR, Detection Rate; BA, Balanced Accuracy.

### Comparison of MASHPredict score with other NITs

A comprehensive comparison was conducted between the MASHPredict score and established NITs, including the Fibrosis-4 Index (FIB-4), AST to Platelet Ratio Index (APRI), AST to ALT Ratio (AAR), and Nonalcoholic Fatty Liver Disease Fibrosis Score (NFS), using ROC curve analysis and DCA (**Supplementary Table 5**). ROC curve analysis revealed that the MASHPredict score achieved the highest AUC for MASH prediction across all cohorts (**Figures 4E–G**). Additionally, DCA demonstrated that the MASHPredict score offered greater clinical net benefit compared to other NITs (**Figures 4H–J**). In conclusion, the MASHPredict score exhibited superior predictive performance for MASH compared to FIB-4, APRI, AAR, and NFS, reinforcing its potential clinical utility.

### Model interpretation

The SHAP framework was employed to interpret the MASHPredict score. The mean absolute SHAP values highlighted the impact of various risk factors, with ACY1 emerging as the most influential variable (**Figures 5A, B**). To further elucidate the interpretability of the MASHPredict score, two representative cases were examined. SHAP values were used to assess the influence of each feature on the predictions made by the MASHPredict score. In this study, reduced SHAP values corresponded to a lower likelihood of MASH, while elevated SHAP values indicated an increased probability. For instance, patient 1, diagnosed with MASH, exhibited an elevated SHAP value and a MASHPredict score of 0.73, suggesting a higher likelihood of MASH (**Figure 5C**). In contrast, patient 2, who did not have MASH, displayed a lower SHAP value and a prediction score of 0.39, indicating a reduced probability of MASH (**Figure 5D**).

**Figure 5:**
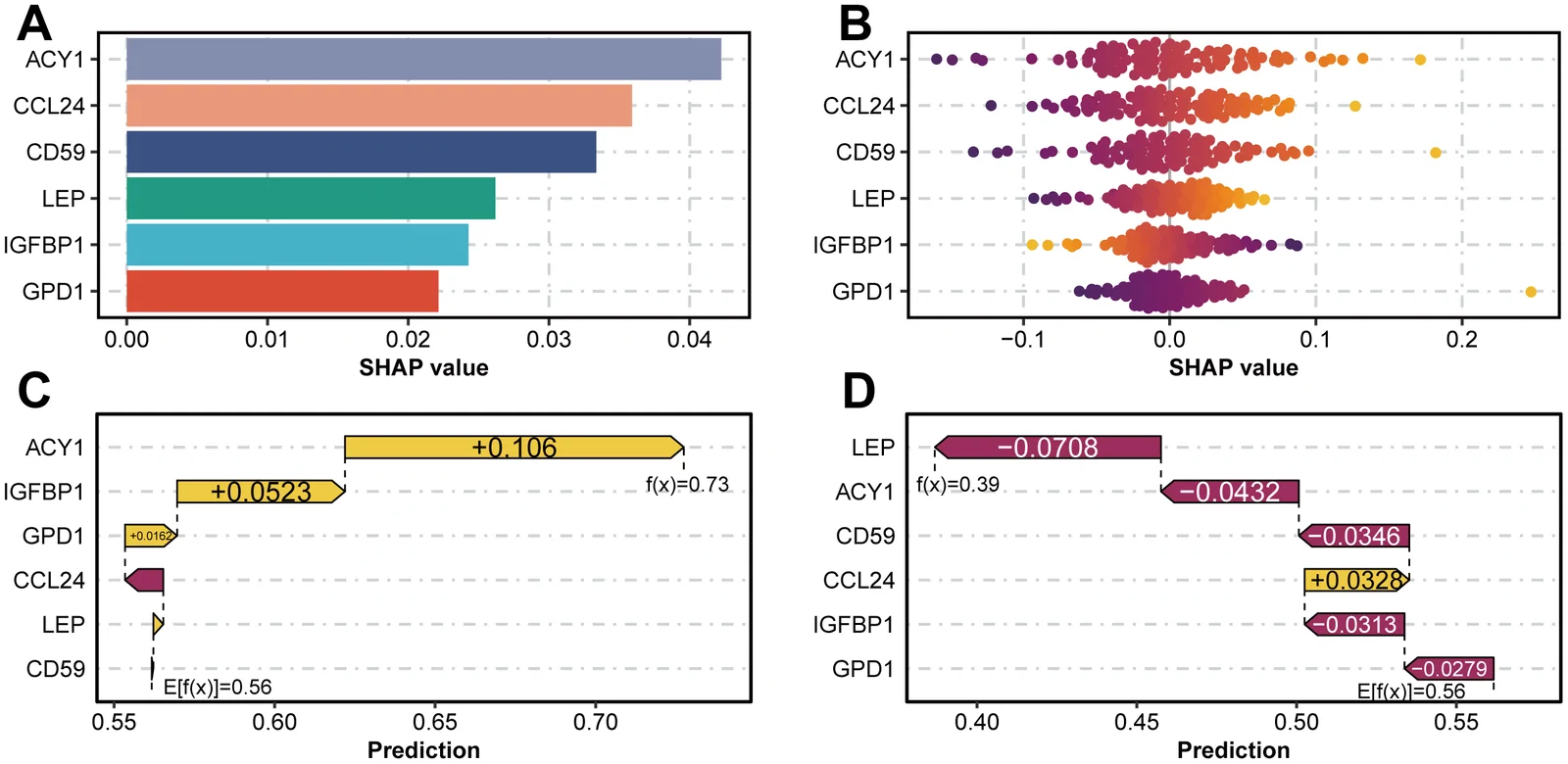
The interpretability of the MASHPredict score. **(A)** Importance ranking of various variables based on the mean (|SHapley Additive exPlanations (SHAP) value|) derived from the MASHPredict score. **(B)** Stability and interpretability of different risk factors assessed with the MASHPredict score. **(C)** SHAP value interpretation in a classical sample with MASH. **(D)** SHAP value interpretation in a classical sample without MASH.

### Prognostic value of MASHPredict score

Participants in the prognostic cohort were divided into low-risk and high-risk groups. For liver-related mortality, the high-risk group demonstrated a significantly increased hazard ratio (HR = 8.18, 95% CI: 3.54-18.90, p < 0.001) (**Figure 6A**). Time-dependent ROC analysis revealed robust prognostic accuracy for liver-related mortality, with AUC values reaching 0.74, 0.78, and 0.79 at 5, 10, and 15 years, respectively (**Figure 6A**). Regarding MASH events, the high-risk group showed substantially elevated risk (HR = 12.07, 95% CI: 4.39-33.18, p < 0.001) (**Figure 6B**). Time-dependent ROC analysis indicated strong prognostic performance, with AUCs of 0.87, 0.85, and 0.83 at 5-year, 10-year, and 15-year follow-up, respectively (**Figure 6B**). For cirrhosis, significantly increased risk was observed in the high-risk group (HR = 3.37, 95% CI: 2.50-4.53, p < 0.001) (**Figure 6C**), with time-dependent AUC values of 0.71, 0.73, and 0.70 at 5, 10, and 15 years, respectively (**Figure 6C**). Concerning liver cancer, the high-risk group exhibited elevated risk (HR = 3.11, 95% CI: 1.43-6.75, p < 0.001) (**Figure 6D**), with time-dependent AUCs of 0.75, 0.74, and 0.72 at 5, 10, and 15 years, respectively (**Figure 6D**). Additionally, the high-risk group demonstrated significantly increased risks for ASCVD (HR = 1.64, 95% CI: 1.39-1.94, p < 0.001) and all-cause mortality (HR = 1.42, 95% CI: 1.33-1.49, p < 0.001) (**Supplementary Figure 2A, B**).

**Figure 6:**
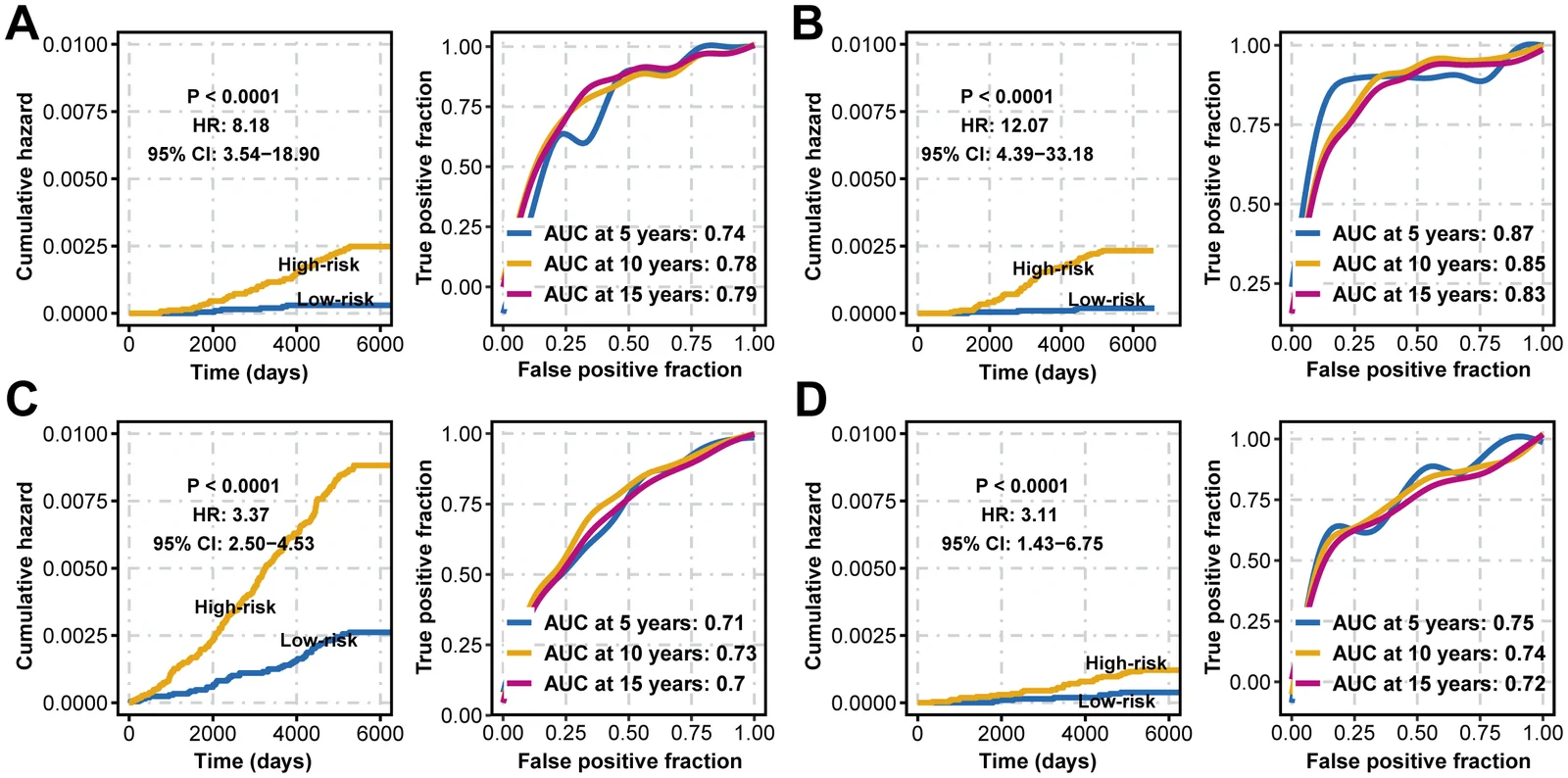
Prognostic value of MASHPredict score in the prognostic cohort. **(A)** Cumulative hazard plot comparing high-risk and low-risk patient groups over time (in days), with liver-related death as the outcome (left). Time-dependent receiver operating characteristic curves of MASHPredict score for predicting liver-related mortality (right). **(B)** Cumulative hazard plot comparing high-risk and low-risk patient groups over time (in days), with MASH as the outcome (left). Time-dependent receiver operating characteristic curves of MASHPredict score for predicting MASH (right). **(C)** Cumulative hazard plot comparing high-risk and low-risk patient groups over time (in days), with cirrhosis as the outcome (left). Time-dependent receiver operating characteristic curves of MASHPredict score for predicting cirrhosis (right). **(D)** Cumulative hazard plot comparing high-risk and low-risk patient groups over time (in days), with liver cancer as the outcome (left). Time-dependent receiver operating characteristic curves of MASHPredict score for predicting liver cancer (right).

### snRNA-seq profiling of the 6 key features in liver tissue

Following analysis of snRNA-seq data from 80, 698 cells, clustering analysis identified 14 distinct cell subclusters. Specific cell types were subsequently annotated using well-established markers (**Supplementary Figures 3A–C**). ACY1, GPD1, and IGFBP1 were predominantly expressed in hepatocytes, while CD59 was mainly expressed in vascular and biliary cells. Notably, CCL24 and LEP showed no detectable expression in the liver tissue snRNA-seq data (**Supplementary Figure 3D**). These results suggest that MASH development may not be solely driven by intrahepatic factors, as some predictive biomarkers appear to originate from extrahepatic sources, indicating potential systemic involvement in MASH pathogenesis.

## Discussion

MASH is a progressive, potentially fatal subtype of metabolic MASLD, for which no approved pharmacotherapy exists and current non-invasive tools remain limited in diagnostic accuracy and risk stratification capacity. Liver biopsy remains the diagnostic gold standard but is restricted by invasiveness, cost, and poor patient acceptability, creating an urgent clinical demand for reliable blood-based biomarkers. In the present study, we developed and validated a plasma proteomics-derived machine learning score, termed MASHPredict, based on six key proteins (ACY1, CD59, CCL24, GPD1, LEP, and IGFBP1) identified via a rigorous machine learning pipeline in a Chinese liver biopsy cohort. The model demonstrated robust diagnostic performance across multi-ethnic cohorts, including Israeli and UK populations, and outperformed established non-invasive tests such as FIB-4. Beyond diagnosis, MASHPredict also showed strong prognostic value for liver-related events, atherosclerotic cardiovascular disease (ASCVD)-related mortality, and all-cause mortality. The favorable performance of MASHPredict may be attributed, at least in part, to the PLS algorithm. PLS addresses this by constructing latent components that maximize the covariance between the protein matrix and the binary outcome, thereby capturing the joint predictive signal rather than relying on individual features in isolation. This property not only enhances predictive stability but also facilitates biological interpretability through variable importance in projection (VIP) scores. Although several proteomics- or machine learning-based MASH models have been proposed recently, MASHPredict offers unique strengths: it is among the few plasma proteomics-based models with transethnic validation and comprehensive prognostic assessment. Notably, our study represents the first plasma proteomics-based MASH diagnostic model to undergo simultaneous diagnostic and long-term prognostic validation, highlighting its incremental novelty and clinical potential.The MASHPredict score provides multiple advantages for MASH diagnosis. First, although this score was initially developed in a Chinese liver biopsy cohort, we validated its robust diagnostic performance in diverse populations, including Israeli and British cohorts. These findings suggest broad applicability of the MASHPredict score across various ethnic groups. Second, plasma proteomics technology, which is becoming increasingly accessible, can measure the components of the MASHPredict score (ACY1, CD59, CCL24, GPD1, LEP, and IGFBP1). Recent developments in proteomics platforms facilitate the integration of the MASHPredict score into routine clinical practice (28). Third, the MASHPredict score was specifically designed and optimized to identify patients with MASH, enabling close monitoring and timely pharmacological intervention. Fourth, the MASHPredict score is applicable to the general population, achieving an NPV of 0.99, which supports its utility in large-scale population-based screening. Fifth, the MASHPredict score demonstrated robust prognostic performance, with AUCs exceeding 0.83 for 5-, 10-, and 15-year MASH event prediction. These findings suggest its potential utility in early risk stratification and targeted interventions, such as dietary counseling and behavioral modifications, for individuals at risk (29).

Clinical context and testing objectives will determine the practical implementation of the MASHPredict score. The AUC values (0.79-0.83) demonstrated in our study suggest that the MASHPredict score effectively identifies patients at high risk for MASH. Patients who test positive for MASH using the MASHPredict score should undergo specialist evaluation for liver stiffness measurement (LSM) through transient elastography and evaluation of potential pharmacotherapeutic options. This sequential strategy, employing MASHPredict for initial MASH identification and LSM for advanced fibrosis evaluation, optimizes the clinical value of these biomarkers and directs appropriate patient care.

Most elements of the MASHPredict score have demonstrated strong individual correlations with MASH. Extensive research has established the association of CD59, CCL24, GPD1, LEP, and IGFBP1 with MASLD or MASH. CD59, a critical membrane complement regulatory protein, primarily functions to protect cells from complement-mediated damage by preventing the formation of the membrane attack complex. In patients with MASH, DNA methylation analysis has shown decreased methylation levels of the CD59 gene, accompanied by significantly elevated mRNA expression levels (25). CCL24, a member of the CC chemokine family, is a pro-fibrotic and pro-inflammatory molecule involved in various chronic inflammatory and fibrotic diseases. Serum CCL24 levels are elevated in patients with MASLD compared to healthy individuals, with higher levels correlating with more severe fibrosis (30). GPD1 is a key enzyme in triglyceride synthesis and the regulation of carbohydrate and lipid metabolism. It catalyzes the conversion of dihydroxyacetone phosphate to glycerol-3-phosphate, providing substrate for triglyceride synthesis. Mutations in GPD1 lead to hepatomegaly, steatohepatitis, and hypertriglyceridemia (31). IGFBP1 regulates insulin-like growth factor activity and plays a role in glucose metabolism, cell proliferation, and inflammatory responses. In *in vivo* experiments, recombinant IGFBP1 treatment in methionine/choline-deficient diet-induced model mice resulted in reduced hepatic inflammation, decreased serum transaminase levels, and markedly suppressed lipogenic gene expression (32). LEP, a 167-amino acid peptide hormone encoded by the obesity gene, is primarily secreted by adipocytes and plays a pivotal role in regulating food intake, energy expenditure, and adiposity (33). LEP’s role in fatty liver disease pathogenesis is complex. In MASH, serum LEP concentrations correlate with hepatic steatosis, inflammation, anthropometric indices, and levels of serum lipids, insulin, glucose, and alanine aminotransferase (34). LEP can also activate hepatic stellate cells, promoting liver fibrosis (35). Collectively, the MASHPredict score incorporates these biomarkers, reflecting the multifaceted pathogenesis of MASH, including inflammation (CD59, CCL24), lipid metabolism (GPD1 and LEP), and glucose metabolism (IGFBP1), among others. To further clarify the biological basis of the MASHPredict model, the six included proteins reflect key pathogenic pathways of MASH. Specifically, CD59 and CCL24 are involved in chronic inflammation and complement activation, which drive hepatic injury and fibrosis. GPD1 and LEP are closely linked to lipid metabolism dysregulation and adipose tissue dysfunction, promoting hepatic steatosis and lipotoxicity. IGFBP1 modulates insulin resistance and hepatic inflammation, while ACY1 participates in amino acid metabolism and redox homeostasis, affecting hepatocyte survival. Collectively, these proteins cover inflammation, lipid metabolism, insulin signaling, and redox balance—core mechanisms underlying MASH pathogenesis. This multi-pathway coverage provides a solid biological rationale for the construction and good performance of the MASHPredict score.

This study has several limitations. First, the external validation using Test cohort 1 (n=57), derived from a public GEO repository, suffers from a small sample size and a lack of most clinical covariates. Consequently, this cohort can only provide preliminary evidence of model generalizability based on proteomic profiles alone, and it should not be regarded as a fully qualified external validation set. Second, Test cohort 2 employed MRI-PDFF combined with cT1 as a surrogate reference standard for MASH instead of liver biopsy. While this approach limits direct comparison with histology, it reflects a pragmatic design for population-based research where invasive biopsy is not feasible. This noninvasive reference standard may lead to slight misclassification, which could modestly attenuate estimated sensitivity and positive predictive value. However, the extremely high negative predictive value (NPV = 0.99) observed in this low-prevalence general population cohort remained robust and supports the clinical utility of MASHPredict for ruling out MASH in large-scale screening. These findings highlight the trade-off between diagnostic gold standard and real-world applicability in translational biomarker studies. Third, the potential of the MASHPredict score for continuous monitoring of hepatic health requires further validation. Additional long-term prospective studies are needed to confirm its predictive accuracy and establish its practical utility in clinical settings for assessing disease progression or improvement following treatment. Furthermore, the present study included data from multiple independent sources, including our in-house clinical cohort, an Israeli cohort, and the UK Biobank. Owing to differences in data collection protocols, measurement platforms, and patient characteristics, integrative pooled analysis across all cohorts was not feasible. In addition, the external validation cohorts rely entirely on publicly available datasets, and we cannot independently verify raw data quality, such as selection bias or measurement error, which may introduce potential confounding. Future prospective, standardized multicenter cohorts are warranted to validate our findings. Notably, although several of the six core proteins are expressed in hepatocytes, their detection in peripheral blood does not confirm a liver-specific origin; contributions from extrahepatic tissues (e.g., adipose tissue, immune cells) cannot be excluded. In addition, these plasma proteins currently lack widely available, conventional quantitative assays in routine clinical practice, which limits immediate large-scale clinical translation of the MASHPredict score. Future development of simple, cost-effective immunoassays will facilitate its broader clinical application.In conclusion, the MASHPredict score is a validated, non-invasive tool applicable to diverse populations. It demonstrates robust predictive ability across various cohorts and effectively assesses the long-term risk of liver-related events. As healthcare systems shift towards managing chronic diseases at the population level, the MASHPredict score offers a scalable approach for early detection and risk stratification. With its adaptability to different healthcare settings (36, 37), the MASHPredict score shows promise for practical implementation and could help alleviate the global burden of MASH.

## Data Availability

The mass spectrometry proteomics data have been deposited to the ProteomeXchange Consortium via the PRIDE partner repository with the dataset identifier PXD085290.

## Supplementary material

Supplementary Figure 1 The process of constructing the MASHPredict score. **(A)** Correlation analysis of 6 key proteins. **(B)** Ranking of the area under the curve (AUC) values for 10 machine learning algorithms evaluated in the validation cohort.

Supplementary Figure 2 Prognostic value of MASHPredict score in the prognostic cohort. **(A)** Cumulative hazard plot comparing high-risk and low-risk patient groups over time (in days), with atherosclerotic cardiovascular disease (ASCVD) as the outcome. **(B)** Cumulative hazard plot comparing high-risk and low-risk patient groups over time (in days), with all-cause mortality as the outcome.

Supplementary Figure 3 Single-nuclei RNA sequencing (snRNA-seq) profiling of the 6 key features in liver tissue. **(A, B)** Visualization of 80, 698 cells using Uniform Manifold Approximation and Projection (UMAP). Clusters were categorized into 37 subtypes **(A)** and 8 major cell types **(B)**. **(C)** Canonical marker genes of major cell types. **(D)** Expression of the 6 key features in liver tissue snRNA-seq.
